# The Relationship Between Perceived Leader Busyness and Perspective Taking and Interaction Behavior of Followers

**DOI:** 10.3389/fpsyg.2021.676810

**Published:** 2021-11-15

**Authors:** Qiufeng Huang, Kaili Zhang

**Affiliations:** ^1^School of Political Science and Public Administration, Huaqiao University, Fujian, China; ^2^School of Business, East China University of Science and Technology, Shanghai, China

**Keywords:** perceived leader busyness, perspective taking, interaction avoidance, performance appraisal, sensemaking theory

## Abstract

How leaders influence followers have been a hot topic in both research and practice. Yet, prior studies have primarily focused on the impact of one leadership style, while overlooking how a leadership role may influence behavioral expressions of leaders. Particularly, being a leader means having to face time demands and workload pressure, and thus, busyness becomes a common phenomenon for leaders. Focused on perceived leader busyness, we had examined how it may influence employee interactions with leaders and how those interactions influenced leader evaluations of the performance of followers. Based on sensemaking theory, we propose that when followers have a high level of perspective taking, they are more likely to take avoidance behavior when perceiving leaders as of high busyness. Further, when followers engage in interaction avoidance behavior, leaders may consider followers as hiding errors or intentionally concealing their work process, which reduces positive evaluations (i.e., task performance and conscientiousness evaluation) while enhancing negative evaluation (i.e., deviance behavior) toward followers. We conducted two studies. Study one was conducted with a 25 participants interview and data of 297 employees to show scale validity of perceived leader busyness. Study two was conducted with 377 employees and their direct supervisors. Applying the complex modeling method, we found that followers with low-level perspective taking are less likely to engage in interaction avoidance behavior, even when perceiving leaders as high busyness; interaction avoidance behavior of followers has a positive relationship with counterproductive behavior evaluation of leaders, but a negative relationship with conscientiousness behavior evaluation. This study enriches the dyadic interactions between leaders and followers. In addition, it also shows the burden of perspective taking.

## Introduction

Being a leader usually means that individuals can have more access to resources. However, it also means that individuals may need to take on more responsibility and handle more challenging tasks (Neal et al., 2017; Sherf et al., 2019). In contemporary workplaces, rapid shifts and changes in the working environment bring about more uncertainties and challenges, which require leaders to accomplish tasks and, at the same time, handle sophisticated problems, make decisions, and solve crises quickly (Jen, 2016). These requirements prompt leaders to deal with a large amount of work in tense schedules. Based on the observation of the daily work of leaders, we realize that leaders not only need to fulfill their routine tasks, such as supervising work progress and task completion of subordinates, guiding and training team members, and coordinating and completing their jobs, but also address emergencies and crises (Jen, 2016; Neal et al., 2017). Therefore, under pressure from numerous work assignments and tight schedules, busyness is a common working status for leaders (Sherf et al., 2019). Therefore, this study focuses on busyness and attempts of leaders to explore their influence on followers.

Previous studies have investigated the concept of busyness and its impact. For example, Bellezza et al. (2016) focused on individual busyness and its impact on self-status satisfaction. Through experimental manipulation, they found that individuals in a busy status gave positive self-evaluations (Bellezza et al., 2016). Wilcox et al. (2016) explored how individual busyness status may influence self-assessment when encountering mission failure. Through experiments, they found that there was a low probability of negative self-assessment due to task failure when individuals were in a busyness status (Wilcox et al., 2016). Although inspiring, the above studies mainly focused on non-workplace contexts and primarily explored the effect on self-assessment. Considering the pervasiveness of busyness in the workplace, recent research began to emphasize busyness in the workplace. For example, Sherf et al. (2019) investigated leader busyness and its influence on the work arrangement of leaders. They discovered that when leaders are busy, they tend to pay more attention to task completion rather than interpersonal relationships (Sherf et al., 2019). However, studies on busyness in the workplace are still limited, which restricts our understanding of leader busyness and how it may have an impact on dyadic interaction between leaders and followers.

It is widely acknowledged that followers pay close attention to the behavior of leaders and are quite sensitive to expressions of leaders. Followers may adjust their interactional modes with superiors by interpreting and analyzing their status. For instance, before reporting their work processes or problems, followers may try to analyze the mood of leaders before deciding to communicate: when followers observe that leaders are in bad mood, they may avoid reporting a problem and reduce their interactions with leaders. When followers consider leaders to be in a good mood, subordinates may increase their interaction frequency with the leader. Hence, the mood of leaders can play a significant role in the interactive behaviors of followers. In this way, when leaders display busyness, followers may form conclusions that may cause them to reduce interactions with said and minimize disturbing those leaders. However, this reaction is a product of the individual characteristics of followers. Following this logic, we argue that if followers assume that they know the feelings of their leaders and understand situations of leaders well, they will reduce their interaction with a busy leader. Therefore, to capture such personal characteristics, we analyze perspective taking.

Perspective taking is defined as the understanding of individuals and consideration of others (Ku et al., 2015; Jing and Baiyin, 2016; Zhang and Tang, 2017). When receiving busyness cues from leaders, followers with high-level perspective taking would have a preference for altruism analysis, assuming that leaders need personal space to handle pressure. They may believe that avoiding interruption is one of the best ways to support a busy leader. For followers with low-level perspective thinking, may lack the ability to understand situations of others and may still interrupt and interact, despite the busyness of leaders. Hence, perspective thinking would work as the key boundary condition between the relationship of leader busyness and interaction avoidance of followers with the leader.

Move beyond, leaders would also interpret the behaviors of followers when making performance appraisals. Leaders often need to evaluate the performance of their followers, and follower behavior provides important signals (Maitlis, 2005; Sandberg and Tsoukas, 2015). When followers convey limited and vague information, leaders may find it difficult to accurately understand the work status of followers (Brown et al., 2015). At the same time, leaders may misinterpret limited information of followers as challenging—or disrespectful (Mead and Maner, 2012; Zheng Y. et al., 2019), which may lead to negative evaluations for followers. To capture the full picture of leaders’ evaluation of followers, we focus on leaders’ task performance evaluation, conscientiousness behavior evaluation, and counterproductive behavior evaluation of followers. This study argues that interaction avoidance behavior of followers may have a negative relationship with leaders’ evaluation of followers’ task performance and conscientiousness behavior, but a positive relationship with counterproductive behavior of followers.

**FIGURE 1:**
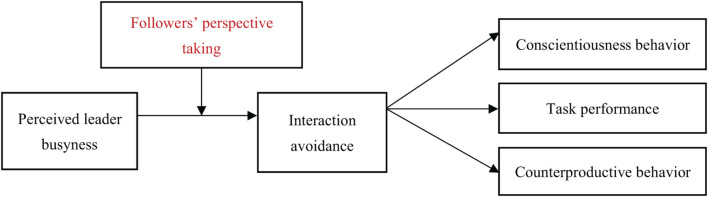
The theoretical model of this study.

Overall, this study makes four contributions to the literature. First, it provides a novel perspective on leadership studies. Studies on leadership generally adopt a leader behavior approach to understanding the outcomes of followers. This study takes a novel perspective to focus on a general image of leaders and their impact on followers. Second, this study enhances and enriches research on busyness. Previous studies have mostly focused on busyness in non-work contexts, with an emphasis on the effects of busyness on individual self-assessment (e.g., Bellezza et al., 2016; Wilcox et al., 2016). This study investigated busyness in organizations and examined how the busyness of leaders may impact dyadic interactions. Correspondently, this study expands and furthers understanding of the impact of busyness. Third, this study contributes to the perspective-taking literature. Literature on perspective taking has mostly focused on its positive impacts, especially on dyadic relationships. Yet, we argue that perspective taking can bring high costs. Followers with high-level perspective taking may receive more negative evaluations than those with low-level perspective taking under certain conditions. Hence, this study enriches perspective-taking research and raises the argument that perspective taking burdens some individuals. Finally, based on the sensemaking theory, this study highlights that not only will followers make sense of the behavior of leaders, but also leaders will make sense of the reactive behavior of followers when making performance appraisals. With limited information, leaders are more likely to make negative rather than positive assessments. Therefore, when faced with uncertain or incomplete information, leaders are inclined to adopt conservative evaluations (Maes et al., 2018; Mai et al., 2018), which further shows the impact of information uncertainty on decisions and performance appraisals of individuals. Figure 1 presents the theoretical model of this study.

## Theory and Hypotheses

### Sensemaking Theory

Sensemaking refers to the process of subjective resolution and the interpretation of external ambiguous and uncertain information by individuals. It generally includes two steps: the construction of external information and subsequent actions (Maitlis, 2005; Weick et al., 2005; Heaphy, 2017; Aguinis and Glavas, 2019). Specifically, in the process of sensemaking, individuals reconstruct meaning in their way based on acquired external information. Definitions with a relatively stable interpretation correspondingly affect behavioral choices (Maitlis, 2005; Heaphy, 2017). There are several core points in the sensemaking theory. First, external information is endowed with unintelligibility, ambiguity, and uncertainty. Contrarily, if the external information is so distinct and explicit that individuals can obtain information directly from it, then there is no demand for reconstruction (Maitlis, 2005; Weick et al., 2005; Heaphy, 2017). Second, during sensemaking, individuals endow realistic meanings by combining information with their existing cognitive paradigm (Aguinis and Glavas, 2019). Thus, individual traits could make a difference. For example, Wrzesniewski et al. (2003) mentioned that when individuals interpret information, they tend to display a confirmation bias, which verifies their existing self-cognition (Wrzesniewski et al., 2003). Third, individuals make decisions in accordance with the consequences of meaning construction (Maitlis, 2005; Weick et al., 2005; Heaphy, 2017), such as changing work content or self-cognition. Confronted with multiple information sources in various workplaces, subordinates prioritize the meaningful construction of important information (Brown et al., 2015). Within an organization, leaders control the allocation of resources and define significant sources of information. Therefore, followers will be sensitive to leadership behaviors and will conduct a systematic interpretation of leadership behaviors (Jiao et al., 2019; Lei and Bing, 2020), which influences their interaction with leaders. In addition, considering that leaders evaluate the performance of followers, they will analyze and judge based on the daily behaviors of followers. Thus, sensemaking exists in leaders and followers, but followers will spend more time and energy (i.e., mobilizing their original cognition) to construct meanings for behaviors of leaders (Weick et al., 2005; Heaphy, 2017), while leaders make relatively direct judgments of the behavior of followers.

### Perceived Leader Busyness

In the contemporary workplace, busyness is a common phenomenon that can be observed in daily greetings among colleagues, complicated work assignments, and limited working time. However, academic research on busyness is limited with most focused on busyness outside the workplace and its influence on self-assessment. For instance, some research mentioned how individual busyness serves as a reminder of spiritual satisfaction, which leads to feelings of psychological pleasure (Hsee et al., 2010; Yang and Hsee, 2019); busyness can increase positive self-evaluation by imposing a sense of task competency on individuals. Busyness can also weaken negative self-evaluation in cases of task delays. In a recent study, leader busyness has been analyzed to explain the working management of leaders (Sherf et al., 2019). Yet, in consideration of the universality of leader busyness in professional settings, it is necessary to deepen our understandings of leader busyness and its corresponding effects on followers.

Different from the research on a specific leadership style (Mengying and Zhenglong, 2018; Jiao et al., 2019; Lei and Bing, 2020), leader busyness reflects the responsibility and pressure brought on leaders by their leadership position. It could be understood that before becoming a leader, individuals may not need to shoulder direct responsibilities, train subordinates, and make strategic decisions. After stepping into a leadership role, individuals will take charge of projects, monitor project progress, undertake responsibilities for the project, and lead and train subordinates (Jen, 2016; Neal et al., 2017). Moreover, a leader must attend a variety of meetings, regular and emergency. Altogether, the work requirements brought about by leadership positions ensure that the leader regularly appears to be busy. However, leadership styles can be chosen freely. For example, leaders may adopt destructive leadership styles when they feel stressed (Mengying and Zhenglong, 2018). In addition, leader busyness and leadership styles can coexist and influence each other. For example, when a leader is too busy, s/he may engage in less interpersonal caring behavior (Sherf et al., 2019), exuding authority, or abusive behavior. Leaders focusing on interpersonal relationships may take compassionate approaches, even when they are busy. Therefore, the busyness and leadership styles of leaders are different but integrated.

Based on busyness research, this study defines leader busyness as confronting high workload and short deadlines, which reflects time pressure and task pressure (Bellezza et al., 2016; Wilcox et al., 2016; Sherf et al., 2019). At the same time, this study emphasizes that perceived leader busyness is the overall busyness status of the leader, without considering the motivation why the leader conveys busyness to their subordinates. Additionally, considering the different work of busy leaders, this study did not distinguish between the content of actually busy leaders, that is, “busy with work” or “busy with private affairs.” In fact, the distinction between motivation and content of busyness may rely more on the judgment of leaders themselves. For example, when the work is finished, the leaders may self-reflect and come to the conclusion that their busyness is ineffective, i.e., “bustle without a plan or purpose.” In terms of busy content, although leaders can share schedules to help followers understand their work, such schedule sharing can also conceal the fact that the leaders are actually busy with private affairs. Hence, it is difficult for followers to differentiate the leader who is “busy with work” from those who are “busy with private affairs.” Therefore, this study merely highlights the judgment of followers of the overall state of the busyness of leaders.

### Perceived Leader Busyness, the Perspective Taking of Followers, and Interaction Avoidance

In workplaces, followers are sensitive to the behavioral expression of their leaders and they fully mobilize their cognitive resources for information interpretation and judgment (Maitlis, 2005; Weick et al., 2005; Heaphy, 2017). Regarding leader busyness, followers with different characters generate different interpretations. Specifically, when followers can interpret the busyness expressed by the leader as the leader being in a stressful environment and in need of more private time, followers engage in less interferential and interactive behaviors. On the contrary, when followers fail to understand the pressure implied upon the busy leader, followers do not reduce their interactions with the leader. Therefore, the ability of followers to understand the situation of leaders can work as an important boundary condition to affect the relationship between perceived leader busyness and the interaction behaviors between followers and leaders.

This study employs perspective taking of followers to capture this character. It is worthy to mention that perspective taking is a vital component of the concept of empathy (Jing and Baiyin, 2016; Zhang and Tang, 2017). Empathy consists of compassion and perspective taking (Grant and Berry, 2011; Zhang and Tang, 2017). Compassion is mainly demonstrated in emotional aspects, which means that individuals can have emotional resonance and empathy for situations of others, especially for unfortunate experiences of others (Zhang and Tang, 2017). Perspective taking is defined as the cognitive part of empathy, referring to a cognitive process in which individuals can accept opinions and attempt of others to comprehend the preferences, values, and needs of others. It also emphasizes that individuals have the ability to explain and understand situations or experiences of others (Grant and Berry, 2011; Zhang and Tang, 2017). Compared with compassion, perspective taking is more often applied to investigate how individuals in organizations understand and analyze the work behaviors of colleagues (Ku et al., 2015). For example, Grant and Berry (2011) found that employees possessing perspective taking were more able to generate ideas beneficial to colleagues (Grant and Berry, 2011). When individuals hold a high-level perspective taking, they spend time observing the behaviors of others; often, they adequately interpret the behaviors of others, understand the situations of others, and take on altruistic behaviors (Grant and Berry, 2011; Ku et al., 2015; Jing and Baiyin, 2016; Zhang and Tang, 2017).

When the leader displays a busy status, followers with a high level of perspective taking tend to interpret leader busyness as the leader needing more time and space to focus on tasks, so they may endeavor to reduce the disturbance toward the leader. In addition, in terms of followers with a high level of perspective taking, they may consider that workload of leaders is already heavy. They hope to reduce additional task pressures on leaders. For example, they solve problems on their own to reduce unnecessary interference with leaders. Therefore, perspective taking could affect the positive relationship between leader busyness and interactive avoidance and enhance this positive relationship under high-level perspective taking. For followers with low-level perspective taking, despite the exhibition of leader busyness, they may insist on completion of their tasks, rather than paying attention to whether the leader is afraid of being disturbed and whether tasks of leaders are finished. As a result, the positive relationship between leader busyness and interactive avoidance may erode under low-level perspective taking. Thus,

H1: The perspective taking of followers moderates the relationship between perceived leader busyness and interaction avoidance. The positive relationship is weakened (vs. enhanced) when followers have low-level (vs. high-level) perspective taking.

### Follower Interaction Avoidance and Leader Appraisal for Followers

Leaders construct meanings based on the behavior of followers and transfer those meanings to performance appraisals of followers. Interaction avoidance behavior of followers could be used as important information to influence evaluations of leaders. To fully comprehend the appraisal of followers by leaders, this study focuses on two aspects of evaluation: positive behavior evaluation and negative behavior evaluation. Negative behavior evaluation is mainly reflected in the evaluation of counterproductive behavior (Bennett and Robinson, 2000; Yang and Diefendoref, 2009), while the positive behavior evaluation has a relatively wide range of content, which can be reflected in the completion of specified tasks and the initiative of employees to undertake additional work. The former is mainly exhibited in the task performance (i.e., followers complete the stipulated content of their work and undertake their responsibilities; Welbourne et al., 1998). However, the initiative to undertake extra work can take on diverse forms, such as voice behavior, helping behavior, taking charge, conscientiousness behavior (Organ, 1997; Farh et al., 2004; Chiaburu et al., 2017; Mengying and Zhenglong, 2018). Considering the fact that this study attempts to show the evaluation results of leaders on interaction avoidance behaviors of followers, typical behaviors are selected as representative. To specify, this study takes conscientiousness behavior – one component of organizational citizenship behavior to show employees taking the initiative to undertake additional work of the organization as the concrete manifestation of this extra-role behavior (Farh et al., 2004).

When the follower adopts interaction avoidance behavior, leaders may lack a specific and detailed understanding of the specific work of the followers, such as the time and effort of followers spent in the work. This may lead to some deviation in the evaluation of followers (Maes et al., 2018; Mai et al., 2018). Considering that the appraisal of followers will directly affect the final performance evaluation result and bonus, leaders may adopt a conservative strategy when making decisions (i.e., they will not make overvalued evaluations that prevent followers from getting overrated evaluation results or a high-performance bonus; Mai et al., 2018). On the other hand, with limited information, leaders are more likely to be suspicious (Maes et al., 2018). For example, the leaders will analyze the lack of interaction with followers may be due to mistakes or slow work progress, which results in negative evaluations. The study also mentions that when there are limited interactions between followers and leaders, leaders will feel that their authority is being challenged and believe that they are not fully respected by followers because they do not receive effective and sufficient work information (Mai et al., 2018). Based on the above inference, leaders may reduce the positive evaluation of followers based on the interactive avoidance behaviors of followers; meanwhile, leaders may produce negative evaluations of followers. As such, interaction avoidance behavior of followers has a negative relationship with evaluations of leaders about task performance and conscientiousness behavior of followers, while a positive relationship between interaction avoidance behavior of followers and the evaluation of leaders about their counterproductive behavior.

H_2__*a*_: Interaction avoidance has a negative relationship with conscientiousness behavior evaluation of leaders toward followers.

H_2__*b*_: Interaction avoidance has a negative relationship with task performance evaluations of leaders toward followers.

H_2__*c*_: Interaction avoidance has a positive relationship with counterproductive behavior evaluation of leaders toward followers.

## Study One: Scale Development and Validation

Study one was to develop and validate the scale of perceived leader busyness following a three-step process. First, we developed items to reflect perceived leader busyness using an interview method. Second, we conducted an exploratory factor analysis (EFA) to test the factor structure of the items. Third, we conducted a series of confirmatory factor analyses (CFA) to examine the convergent and discriminant validity of the scale of perceived leader busyness.

### Interview

Since the concept of perceived leader busyness is still rarely studied and has not been explored in the Chinese context, we conducted an interview before the questionnaire survey. The sample for the interview was composed of 25 frontline staff members from three manufacturing and service companies located in Shanghai, Shandong, and Jiangsu provinces. Among the samples, 15 were female, and the average tenure was 3.65 years. All participants had a high school education degree or above. Each recorded interview lasted approximately 30 min.

We asked three questions during the interviews. Question one asked whether followers can perceive the busyness of a leader. This question aimed to verify whether leaders will convey messages about their busyness to their followers. Question two is “what are the specific behavioral manifestations of leaders’ busyness?” This question aimed to explore the behavioral manifestations of leader busyness. Question three is “what do followers do after perceiving leader busyness?” This question was attempted to depict the effect of the busyness of leaders on the behavior of followers. As shown in Table 1,92% of participants reported that followers could perceive leader busyness, while the other 8% of participants had difficulty perceiving the busyness of the leader because of limited contact with leaders. As for specific behavioral manifestations of leader busyness, 96% of participants reported that leaders have more responsibility for work, 88% of participants perceived leader busyness by verbal communication of leaders, 80% of participants recognized that leaders have to deal with urgent tasks, and 48% of participants perceived leader busyness because leaders shared their tight schedules. Furthermore, 96% of participants tried not to communicate with the leader, out of concern over disturbing supervisors. Another 60% of participants indicated that communicating with the leader was very necessary, even if the leader was busy.

**TABLE 1:** Interview results.

Question	Percentage
**Question one: Can followers perceive the busyness of a leader?**	
1. Absolutely, yes	23/25 (92%)
2. It is hard to perceive the busyness of a leader	2/25 (8%)
Question two: What are the specific manifestations of leader busyness?	
1. Leader verbally communicates to us that s/he is busy	22/25 (88%)
2. Leader shows work schedule to prove busyness	12/25 (48%)
3. Subordinate recognize that leader = deals with very urgent tasks	20/25 (80%)
4. Intensive work schedule	20/25 (80%)
5. Leader takes more responsibility for work	24/25 (96%)
**Question three: What do followers do after perceiving leader busyness?**	
1. Try not to communicate with the leader, out of concern over disturbing supervisors.	24/25 (96%)
2. Seeking help from colleagues, if necessary	14/25 (56%)
3. Deal with the difficulty or problems alone	20/25 (80%)
4. Communicate important issues to the leader even though s/he is busy	15/25 (60%)
5. Communication with the leader is necessary, whether the leader is busy or not	18/25 (72%)

Interview results provide preliminary support for the theoretical model of our study. In addition, since previous literature on busyness mainly adopted experimental manipulation (Bellezza et al., 2016; Wilcox et al., 2016) and did not provide a specific measurement scale for perceived leader busyness, we thus developed a measurement scale of perceived leader busyness. Combined with previous experimental manipulation of leader busyness and interview results, our study proposed that perceived leader busyness can be manifested in three ways: (1) the time pressure of work; (2) the size of the workload; and (3) whether the leader expresses his/her busyness directly. As showed in Table 2, the study proposed a four-item scale of perceived leader busyness. The four items were as follows: “My supervisor has a lot of work to do,” “My supervisor has a very intense work schedule,” “My supervisor expressed to me that he/she is busy,” and “My supervisor tells me that he/she is busy.”

**TABLE 2:** Exploratory factor analysis.

Items	Factor loading	
1. My supervisor has a lot of work to do	0.833	
2. My supervisor has a very intense work schedule	0.771	
3. My supervisor expressed to me that he/she is busy	0.855	
4. My supervisor tells me that he/she is busy	0.812	
Explanatory power		66.96%
Reliability		0.832

### Scale Validation

Based on the four items above, the reliability and validity of the scale were further verified by a survey. We invited three Ph.D. students and one professor majoring in organizational behavior, who was not involved in this study, to evaluate the content validity of the scale. The inter-rater consistency reliability for this measure was 0.961, indicating that the four items of scale can be used to measure perceived leader busyness.

An EFA was conducted to verify the convergent validity of the scale. Data were collected from a manufacturing company located in Shangdong Province. A total of 297 valid questionnaires were received (a 99% response rate). Of all participants, 32.56% were females. An average tenure was 1.36 years (SD = 1.223), and the average age was 23.34 (SD = 2.512). The EFA results are presented in Table 2. We adopted principal component analysis with varimax rotation. Based on eigenvalues greater than 1-factor analysis, EFA yielded a single factor (explaining 66.96% of the total variance). The factor loading of all individual items ranged from 0.771 to 0.855, with an average factor loading of 0.818. The reliability coefficient of perceived leader busyness was 0.832.

We also tested the convergent validity and discriminant validity of the scale of perceived leader busyness using CFA through *Mplus* 7.0. Considering that leader busyness also implicates that leaders may be difficult to contact and have limited support for followers, we propose that perceived leader busyness is positively correlated with perceived uncertainty of management style. Perceived uncertainty of management style was assessed with a three-item scale developed by Thau et al. (2009). The sample item was “I find management’s actions and decisions unpredictable.” Moreover, leader busyness reflects that the leader has a lot of work to do, which is related to the effectiveness of leaders (Zheng Y. et al., 2019). We propose that perceived leader busyness positively relates to perceived leadership effectiveness. The perceived leadership effectiveness scale was assessed using five items developed by Van Knippenberg and Van Knippenberg (2005). The sample items were “My supervisor is an excellent supervisor.” In addition, certain leadership styles may influence the perception of followers about leader busyness. For example, authoritarian leadership leads to less contact between followers and leaders, which, in turn, increases perceived leader busyness of followers. Hence, we propose a positive relationship between authoritarian leadership and perceived leader busyness. The authoritarian leadership scale was assessed by nine items developed by Zheng Y. et al. (2019). The sample item was “My supervisor requires me to follow his/her instructions completely.”

The measurement models were estimated using the maximum-likelihood procedure. The model fit indices are presented in Table 3. The results showed that the hypothesized model fits the data well: χ*^2^* (183) = 472.04, *p* < 0.01, comparative fit index (CFI) = 0.932, standardized root mean square residuals (SRMR) = 0.067, and the root mean square error of approximation (RMSEA) = 0.072^1^. Moreover, the four-factor model was superior to the other alternatives examined. For example, a comparison of the three-factor model combining perceived leader busyness and perceived uncertainty of management style, using the chi-square difference test [Δχ*^2^* (3) = 482.93, *p* < 0.001]. These results support the distinction between the perceived leader busyness scale and other measurements.

**TABLE 3:** Confirmatory factor analysis.

Models	χ*^2^*	*df*	Δχ*^2^*	Δ*df*	RMSEA	CFI	SRMR
Four -factor model (Hypothesized model)	472.043	183	–	–	0.072	0.932	0.067
Three-factor model (Combing perceived leader busyness and perceived uncertainty of management style)	954.973	186	482.930**	3	0.123	0.813	0.102
Three-factor model (Combing perceived leader busyness and leadership effectiveness	832.046	186	360.003**	3	0.109	0.843	0.101
Three-factor model (Combing perceived leader busyness and authoritarian leadership)	1400.656	186	928.613**	3	0.145	0.712	0.198
Two-factor model (Combing perceived leader busyness and authority leadership; combing leadership effectiveness and perceived uncertainty of management style, respectively)	1884.978	188	1412.935**	5	0.167	0.589	0.209

N = 297. *p < 0.05. **p < 0.01.

Table 4 presents the results of means, SDs, and correlations among demographic variables, perceived leader busyness, perceived uncertainty of management style, leadership effectiveness, and authoritarian leadership. Perceived leader busyness was positively associated with the perceived uncertainty of leader management style (*r* = 0.311, *p* < 0.01). Meanwhile, perceived leader busyness was positively associated with leadership effectiveness (*r* = 0.373, *p* < 0.01), but was not significantly correlated with authoritarian leadership (*r* = 0.092, *p* > 0.05). The correlation results indicated that perceived leader busyness has a moderate or small correlation with other variables, which verifies that perceived leader busyness in this study has good convergent validity and discriminant validity.

**TABLE 4:** The correlations between perceived leader busyness and related variables.

Variables	Mean	SD	1	2	3	4	5	6	7	8
1.Age	23.342	3.512								
2.Tenure	1.362	1.216	0.351**							
3.Gender	0.331	0.468	–0.003	–0.167						
4.Education level	2.499	1.036	0.162**	0.056	0.078					
5.Percived leader busyness	3.628	0.637	0.082	–0.041	−0.211**	0.032	(0.832)			
6. Perceived uncertainty of management style	3.379	0.643	–0.083	0.022	–0.032	0.014	0.311**	(0.889)		
7.Leadership effectiveness	4.032	0.671	0.019	–0.056	–0.045	0.082	0.373**	0.441**	(0.945)	
8. Authoritarian leadership	2.703	0.631	0.107	0.012	−0.122*	0.037	0.092	-0.078	−0.202**	(0.893)

N = 297. Gender: 0 = Male, 1 = Female. Coefficients alfa are displayed on the diagonal. *p < 0.05. **p < 0.01.

## Study Two: Hypotheses Testing

### Sample and Procedure

The sample for study two was composed of technician employees working in manufacturing from three companies located in Jiangsu Province. Employees are grouped into teams, and each team has a formal leadership supervisor. Data were collected from two sources. First, employees were asked to assess their demographic information, perceived leader busyness, perspective taking, and interaction avoidance. Second, leaders were asked to evaluate conscientiousness behavior, task performance, and counterproductive behavior of subordinates^2^. During the survey, we assured the participants that the research was only for academic purposes, and there were no right or wrong answers. We also informed them of the confidentiality of their responses and that their personal information would be removed from the dataset at the completion of the study. All participants were informed that their participation was voluntary.

To ensure an independent evaluation, leader and employee participants were invited to fill out the questionnaires in different offices. Based on work identification, we later paired the supervisor-subordinate questionnaires. The survey was completed by 520 subordinates. Of these, questionnaires of 377 subordinates were matched with the evaluations of 57 supervisors, resulting in 72.5% response rates. The final participant sample had an average age of 40.473 (SD = 9.376), 57.326% had tenure of 5–8 years, 32.212% had tenure of 3–5 years, while the remaining has less than 3 years in the company. Overall, 56.831% of the participants are women.

### Measures

All measures except for the measure of perceived leader busyness were adopted from the original English-language scale. The survey instrument was administered in Chinese. All original scales developed in English underwent a back-translation process. All items were first translated into Chinese by a management professor and then translated back into English independently by another management scholar. Further, one bilingual management professor compared the two versions of the scales and made modifications to resolve the discrepancies. All items of perceived leader busyness and interaction avoidance were rated on a 5-point Likert scale ranging from 1 (“strongly disagree”) to 5 (“strongly agree”). The scales of perspective taking, conscientiousness behavior, task performance, and counterproductive behavior of employees were rated on a 7-point Likert scale ranging from 1 (“strongly disagree”) to 7 (“strongly agree”).

Perceived leader busyness: Subordinates rated the scale of perceived leader busyness using the four items developed in study one. The sample item is “My supervisor has a lot of work to do.” The reliability coefficient of the perceived leader busyness in this sample was 0.843.

#### Perspective Taking

Davis (1980) instrument for measuring perspective taking was adopted in this study. The original scale consisted of seven items, such as two reverse-scoring items. The two reverse-scoring items are: “I sometimes find it difficult to see things from the ‘other person’s’ point of view.” “If I’m sure I’m right about something, I don’t waste much time listening to other people’s arguments.” The reliability coefficient of perspective taking was 0.513, such as these two reverse-scoring items. To increase the reliability of the scale, these two reverse-scoring items were deleted. Thus, five items were used in study two. A sample item was “When I’ m upset with someone, I usually try to ‘put myself in their shoes’ for a while.” The reliability coefficient of perspective taking was 0.666.

#### Interaction Avoidance

Interaction avoidance was assessed using the four-item scale developed by Nifadkar et al. (2012). The original scales of interaction avoidance consisted of eight items. Four items emphasized the negative side of interaction avoidance. The sample items were “Stay away from the supervisor’ is my policy” and “I avoid initiating contact with my supervisor.” Four items were deleted because of the concern that we focused on the neutral part of interaction avoidance. Therefore, four items were used in our study. The items were as follows: “I try to minimize official interactions with my supervisor.” “As much as possible, I do not ask for help or information from my supervisor.” “I prefer having minimal informal interaction with my supervisor.” “I try to have purely official, business-like, interaction with my supervisor.” The reliability coefficient of interaction avoidance was 0.825.

#### Conscientiousness Behavior

Conscientiousness behavior was measured using Farh et al. (2004) four-item organizational citizenship behavior scale. Items were “cooperation at work,” “work overtime,” “activism at work,” and “information sharing.” The reliability coefficient of this scale was 0.853.

#### Task Performance

Task performance was assessed with the four-item scale developed by Welbourne et al. (1998). Task performance is referred to doing things specifically related to one’s job description. Items were “quantity of work output,” “quality of work output,” “accuracy of work,” and “customer service provided (internal and external).” The reliability coefficient of this scale was 0.920.

#### Counterproductive Behavior

We assessed counterproductive behavior with six items adapted from Bennett and Robinson (2000) and Yang and Diefendoref (2009). The sample item was “[This employee] intentionally worked slower.” The reliability coefficient of this scale was 0.927.

#### Control Variables

Several variables were included as control variables, such as age, gender, tenure, and educational level of the subordinates. We also controlled the company for data collection from three different companies. We controlled for leader-member exchange (LMX) because previous literature indicated that LMX is related to the interaction between subordinates and supervisors and task performance of employees. The LMX variable was measured using seven items from Joseph et al. (2011). The sample item of LMX was “My supervisor understands my job problems and needs.” The reliability coefficient of the LMX was 0.832. In addition, we controlled the traditional values of followers because they may affect their responses to perceived leader busyness. The traditional value variable was measured using nine times from Chinese traditional culture scales. The sample items included “loyalty to supervisors,” “ordering relationships by status, and observing this order” (Chinese Cultural Connetion, 1987). The reliability coefficient of the traditional values was 0.915.

### Analytic Strategy

Study two invited one supervisor to evaluate conscientiousness behavior, task performance, and counterproductive behavior of six subordinates. Given that employees were nested with supervisory groups, we used a sandwich estimator to account for the clustering effect by including the syntax TYPE = COMPLEX in the *Mplus* 7.0 (Liu et al., 2015). This method corrects the potential bias in estimation that results from data non-independence because employees are clustered within groups.

### Preliminary Analysis

We first conducted a one-way random-factor analysis of variance. Results showed that the between-person variances were significant for behavior variables of subordinates. Specifically, the between-persons variances were significant for conscientiousness behavior, intraclass correlation coefficient, ICC(1) = 0.504, *F* (41, 335) = 10.13, *p* < 0.01; task performance, ICC(1) = 0.683, *F* (41, 335) = 20.37, *p* < 0.01; and counterproductive behavior, ICC(1) = 0.657, *F* (41, 334) = 18.19, *p* < 0.01. Thus, there were substantial variances in the dependent variables at the between-person level, requiring the use of multilevel modeling for data analysis.

To show the common method variance, we firstly performed Harman’s (1976) one-factor test by means of unrotated principal-component factor analysis. The analysis extracted six factors with eigenvalues greater than 1; the first factor accounted only for 29.7% of the variance. We interpret the absence of a single factor that accounts for most of the variance as evidence that common method variance poses no serious problem. Second, following Podsakoff et al. (2003), we controlled for the influence of an unmeasured latent method factor. We conducted a CFA in Mplus 7.4 and allowed all items to load on their respective construct and a latent method factor. The model fits the data well (χ^2^ = 243.50; *p* < 0.001; *df* = 155; χ^2^/*df* = 1.57; CFI = 0.96; Tucker-Lewis Index (TLI) = 0.95; SRMR = 0.07; RMSEA = 0.05). Yet, the more parsimonious model, in which all items loaded only on their respective construct, showed a better fit [χ^2^ difference (*df* = 1) = 11.02; *p* < 0.001]. Overall, the findings indicate that common method bias is not a significant problem in this study.

We conducted a series of CFAs to examine the convergent and discriminant validity of the measurement model. A six-factor model was examined by loading items on their respective latent variables. As shown in Table 5, the six-factor model fits the data quite well, χ^2^ (309, *N* = 334) = 818.121, *p* < 0.01, RMSEA = 0.067, CFI = 0.918, SRMR = 0.075. In addition, the six-factor model fits significantly better than the other models. For example, the five-factor model combining perceived leader busyness and interaction avoidance, χ^2^ (314, *N* = 334) = 1337.18, RMSEA = 0.094, CFI = 0.835, SRMR = 0.129, fits significantly worse than the six-factor model and five-factor models, Δχ^2^ (5, *N* = 334) ≥ 242.52, *ps* < 0.01. We also examined the four-factor model [Δχ^2^ (9, *N* = 334) = 759.893, *ps* < 0.01], three-factor model [Δχ^2^ (12, *N* = 334) = 1761.840, *ps* < 0.01], and the two-factor model [Δχ^2^ (14, *N* = 334) = 2247.813, *p* < 0.01]. These model comparison results show that the measures captured distinct constructs. Moreover, the factor loading of all perceived leader busyness items was 0.592–0.913, indicating that the measures had good convergent validity.

**TABLE 5:** Confirmatory factor analysis.

Models	χ*^2^*	*df*	Δχ*^2^*	Δ*df*	RMSEA	CFI	SRMR
Six-factor model (theoretical model)	818.121	309	–	–	0.067	0.918	0.075
Five-factor A	1337.176	314	519.055**	5	0.094	0.835	0.129
Five-factor model B	1060.673	314	242.552**	5	0.081	0.88	0.095
Five-factor model C	1398.122	314	580.001**	5	0.097	0.825	0.128
Four-factor model D	2346.577	318	1528.456**	9	0.132	0.673	0.146
Four-factor model E	1578.014	318	759.893**	9	0.104	0.797	0.142
Three-factor model F	2834.727	321	2016.606**	12	0.146	0.595	0.179
Three-factor model G	2579.961	321	1761.840**	12	0.139	0.636	0.156
Two-factor model H	3065.934	323	2247.813**	14	0.152	0.558	0.187

N = 377. *p < 0.05. **p < 0.01.

*Five-factor A model combing perceived leader busyness and interaction avoidance; Five-factor model B combing perceived leader busyness and perspective taking; Five-factor model C combing conscientiousness behavior and task performance; Four-factor model D combing variables conscientiousness behavior, task performance, and counterproductive behavior; Four-factor model E combing variables perceived leader busyness, perspective taking, and interaction avoidance; Three-factor model F combing variables conscientiousness behavior, task performance, and counterproductive behavior, and combing perceived leader busyness and interaction avoidance; Three-factor model G combing perceived leader busyness, perspective taking, and interaction avoidance, and combing perceived leader busyness and perspective taking; and Two-factor model H combing variables subordinates and leader rated separately. RMSEA, root mean square error of approximation; CFI, comparative fit index; and SRMR, standardized root mean square residuals.*

Table 6 presents the means, SDs, and correlations of these variables. The results showed that perceived leader busyness was not significantly correlated with interaction avoidance (*r* = − 0.045, *p* > 0.050); interaction avoidance of followers was negatively correlated with conscientiousness behavior evaluation of leaders (*r* = − 0.324, *p* < 0.01); interaction avoidance of followers was positively correlated with counterproductive behavior evaluation of leaders (*r* = 0.243, *p* < 0.01), and interaction avoidance of subordinates was not significantly correlated with task performance evaluation of leaders (*r* = − 0.047, *p* > 0.05).

**TABLE 6:** Means, SDs, correlations, and reliabilities among the focal variables.

Variable	Mean	SD	1	2	3	4	5	6	7	8	9	10	11	12	13	14	15
1.Company 1	0.255	0.436															
2.Company 2	0.257	0.438	−0.344**														
3.Company 3	0.239	0.427	−0.327**	−0.330**													
4.Age	40.474	9.376	0.285**	−0.129*	−0.585**												
5.Gender	0.430	0.512	0.341**	−0.446**	−0.159**	0.479**											
6.Tenure	3.350	1.501	0.394**	0.132*	−0.509**	0.600**	0.218**										
7.Education	1.330	0.659	−0.263**	−0.274*	0.763**	−0.592**	–0.101	−0.530**									
8. LMX	3.602	0.552	0.058	−0.407**	0.106*	0.074	0.146**	–0.085	0.086	(0.832)							
9.Tranditional values	7.241	1.467	−0.207**	0.337**	−0.240**	0.011	−0.152**	0.137*	−0.140**	–0.078	(0.915)						
10.PLB	3.476	0.908	−0.285**	0.024	0.060	–0.097	–0.090	−0.139**	0.043	0.162**	0.093	(0.843)					
11.Perspective taking	5.362	0.778	0.037	0.148**	0.031	−0.142**	–0.090	0.004	0.058	0.020	0.082	0.004	(0.666)				
12.Interaction avoidance	3.150	0.831	0.045	0.283**	−0.470**	0.233**	–0.006	0.228**	−0.355**	−0.160**	0.096	–0.045	0.006	(0.825)			
13.CB	5.353	0.920	0.471**	−0.333**	0.095	–0.047	0.194**	0.058	0.116*	0.107*	−0.135*	–0.044	0.068	−0.324**	(0.853)		
14.Task performance	4.288	0.770	0.074	−0.215**	0.094	0.007	0.119*	0.009	0.122*	0.200**	–0.059	–0.071	0.111*	–0.047	0.386**	(0.920)	
15.CWB	1.683	0.849	−0.457**	0.031	0.106*	–0.038	−0.135**	−0.199**	0.115**	0.039	0.129*	0.111*	−0.130*	0.243**	−0.473**	−0.030	(0.927)

*N = 377. Companies were dummied coded. Gender: 0 = Male, 1 = Female. Coefficient alfas are displayed on the diagonal.*

*PLB, Perceived leader busyness; CB, Conscientiousness behavior; and CWB, Counterproductive behavior.*

*^∗^p < 0.05. ^∗∗^p < 0.01.*

### Hypotheses Testing

Table 7 presents the unstandardized coefficient estimates for the model. The results showed that predictors included in the model accounted for 39.1% of the total variance in conscientiousness behavior, 9.6% of the total variance in task performance, and 41.2% of the total variance in counterproductive behavior. The results indicated that the model explained a sizable percentage of the variance in the dependent variables.

**TABLE 7:** Unstandardized regression coefficients.

Variables	Interaction avoidance			Conscientiousness behavior			Task performance			Counterproductive behavior		
	Model 1	Model 2	Model 3	Model 1	Model 2	Model 3	Model 1	Model 2	Model 3	Model 1	Model 2	Model 3
Intercept	4.599**	4.619**	4.911**	4.535**	4.545**	6.079**	3.199**	3.093**	2.816**	2.584**	2.557**	1.413
Company 1	–0.213	−0.259*	–0.264	1.092**	1.107**	1.023**	–0.149	–0.146	–0.131	−1.093**	−1.103**	−1.041**
Company 2	0.034	0.014	–0.007	–0.188	–0.182	–0.179	–0.296	–0.290	–0.291	−0.466*	−0.469*	−0.471*
Company 3	−0.913**	−0.931**	−0.893**	0.104	0.112	–0.196	–0.280	–0.294	–0.238	–0.299	–0.307	−0.077*
Age	–0.001	–0.002	0.000	–0.015	–0.015	−0.015*	–0.005	–0.006	–0.006	0.012*	0.012*	0.012*
Gender	–0.046	–0.046	–0.068	0.135	0.134	0.118	0.074	0.082	0.085	–0.214	–0.211	–0.200
Tenure	0.011	0.012	0.018	0.031	0.031	0.034	0.072	0.069	0.069	–0.016	–0.016	–0.019
Education	–0.074	–0.081	–0.062	0.184	0.187	0.161	0.241	0.235	0.239	0.037	0.034	0.053
LMX	–0.177	–0.167	–0.177	0.015	0.011	–0.045	0.202	0.206	0.216	–0.026	–0.023	0.019
Traditional values	–0.058	–0.058	–0.061	0.009	0.008	–0.011	–0.026	–0.025	–0.021	–0.014	–0.014	0.001
Perceived leader busyness (PLB)		–0.048	–0.061	0.077	0.091	0.077	–0.088	–0.082	–0.080	–0.029	−0.038*	–0.028
Perspective taking (PT)		0.028	–0.028	0.071	0.059	0.067	0.119	0.139	0.138	–0.100	–0.088	–0.094
PLB*PT			0.242**		0.013	0.016		–0.086	–0.087		–0.025	–0.027
Interaction avoidance						−0.332**			0.060			0.248**
*R* ^2^	0.258	0.261	0.305	0.344	0.345	0.391	0.092	0.094	0.096	0.378	0.378	0.412
Δ*R*^2^	0.258**	0.003	0.044**	0.344**	0.001	0.046**	0.092**	0.002	0.002	0.378**	0.000	0.034**

N = 377. *p < 0.05. **p < 0.01. Unstandardized regression coefficients are shown in the diagonal.

**FIGURE 2:**
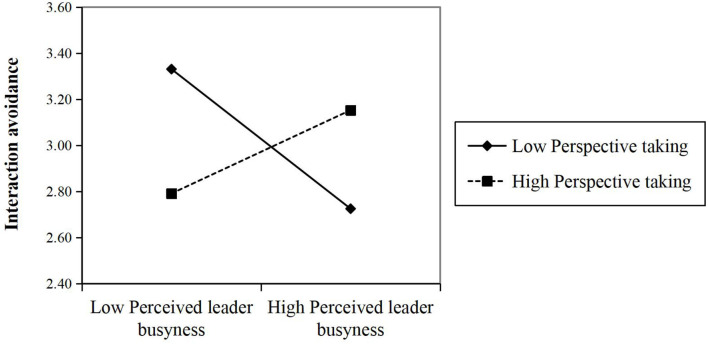
The moderating role of perspective taking of followers on the perceived leader busyness: interaction avoidance relationship.

Hypothesis 1 proposed that perspective taking of followers moderates the relationship between perceived leader busyness and interaction avoidance. As expected, the results of Model 3 in Table 7 indicate that although the main effect of perceived leader busyness had no significant effect on interaction avoidance (γ = − 0.061, *p* > 0.05), the interaction term of perceived leader busyness and perspective taking of followers was significantly related to interaction avoidance (γ = 0.242, *p* < 0.01). We plot this positive moderating effect in Figure 2. Simple slope analyses indicated that perceived leader busyness was negatively related to interaction avoidance (γ = − 0.249, *p* < 0.01) with low followers’ perspective taking (1 SD below the mean). When the perspective taking of followers was high (1 SD above the mean), the relationship became non-significant (γ = 0.125, *p* > 0.10). Overall, the difference in relationship magnitude of perspective taking between the high and low followers was significant (γ = 0.374, *p* < 0.01). Hence, Hypothesis 1 was partially supported, indicating that followers with low-level perspective taking are less likely to engage in interaction avoidance behavior, even when perceiving leaders as busy.

Hypothesis 2 proposed that interaction avoidance influences conscientiousness behavior, task performance, and counterproductive behavior. After controlling for LMX and traditional values, interaction avoidance was negatively related to conscientiousness behavior evaluation of leaders (γ = − 0.332, *p* < 0.01) and positively related to counterproductive behavior evaluation of leaders (γ = 0.248, *p* < 0.01). Hence, Hypotheses 2_*a*_ and 2_*c*_ are supported. However, interaction avoidance was not related to task performance evaluation of leaders (γ = 0.060, *p* > 0.05), providing no support for Hypothesis 2_*b*_.

**FIGURE 3:**
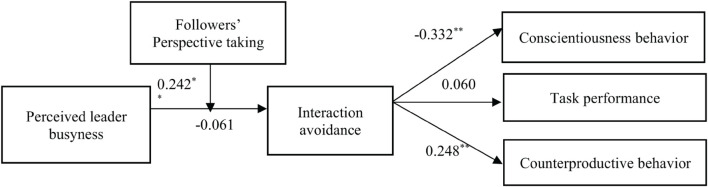
Results of the hypothesized model. Note: *N* = 377, unstandardized estimates of the path coefficients, ^∗^*p* < 0.05, ^∗∗^*p* < 0.01.

Although the indirect effect between variables was not hypothesized in this study, we also performed an analysis using the Monte Carlo method (Selig and Preacher, 2008) to estimate the indirect effects. With 2,000 Monte Carlo replications, the results showed that perceived leader busyness interacts with perspective taking to have an impact on conscientiousness behavior via the mediating effects of interaction avoidance, γ = − 0.083, 95% CI = [−0.1394, −0.0322] and counterproductive behavior evaluation, γ = 0.058, 95% CI = [0.0179, 0.1105]. The CIs did not contain zero, indicating that the interaction effect of perceived leader busyness and perspective taking was positively and significantly related to conscientiousness behavior and counterproductive behavior via interaction avoidance. The results of the hypothesized model are shown in Figure 3.

## Discussion

From the perspective of sensemaking, this study focuses on the phenomenon of leader busyness and explores its influence on the interaction between followers and leaders. Emphasis is placed on the influence of the perspective-taking traits of followers in these relationships. In addition, the behavior evaluation of followers by leaders based on the interactive avoidance behavior of subordinates is further explored. In the first study, through interviews and data collected from 297 employees, the influence of perceived leader busyness in the organization and the response of followers were preliminarily understood. The reliability and validity of the perceived leader busyness scale were developed and verified. Based on the data analysis of 377 followers and their direct superiors, the second study found that compared with followers with high-level perspective taking, the followers with low-level perspective taking did not engage in interaction avoidance when faced with busy leaders. In this regard, we concluded that low-level perspective-taking followers considered their situation more important than high-level perspective-taking followers. The low-level perspective-taking followers will worry about their issues and try to seek the support of leaders despite the situation of leaders, yet this is not the case for followers with high-level perspective taking. However, we did not find a positive relationship between perceived leader busyness and interaction avoidance behavior of followers. We contend that this may be because that followers may also take account of the task characteristics when deciding their interaction with the leader: when the task is of great importance and urgency, even followers have a high-level perspective taking, they may also need to contact with leaders. Hence, different contextual factors may also impact the interaction avoidance behavior of employees with busy leaders. It is also found that leaders construct meaning according to the behaviors of their followers; that is, when followers exhibit interactive avoidance behaviors, they will decrease their evaluation of positive behaviors (i.e., conscientiousness behavior) and increase their evaluation of negative behaviors (i.e., counterproductive behaviors) of their followers. However, the study did not find a relationship between the interactive avoidance of followers and the evaluation of leaders of their task performance behavior. This finding indicates that the interactive avoidance behavior may have more impact on the evaluation of leaders of the extra-role behavior of subordinates.

### Theoretical Contributions

This study makes four major contributions to this theory. First, this study provides a new perspective for the study of leader behavior. Previous studies mostly focused on a specific leadership style, while the busyness of leaders in this study was tightly connected with the leadership positions. When taking on a leadership role, completing the work content required by the position and taking on more responsibilities are necessary and indispensable. Thus, the leadership position will directly create a busy status. However, research on leadership style expresses the independent choice of leaders with featured styles and personal characteristics. For example, proactive personality affects the choices of leaders of specific leadership styles (Lam et al., 2018). Perceived sense of leaders of pressure or time urgency may lead to different leadership styles (Mengying and Zhenglong, 2018). In addition, leadership busyness and leadership styles are not exclusive but compatible. For example, busy leaders may adopt a negative leadership style, such as abusive behavior or authoritative leadership behavior (Mengying and Zhenglong, 2018; Zheng Y. et al., 2019). Similarly, leaders can show benevolent leadership even when they appear busy. Therefore, research on leader busyness provides a new perspective for research on leader behavior.

Second, this study provides a new method for penetrating busyness. Although a few previous studies have focused on busyness, existing studies mainly focused on an individual’ busyness and its influence on the individual’s self-evaluation (Hsee et al., 2010; Bellezza et al., 2016; Wilcox et al., 2016; Yang and Hsee, 2019). They paid less attention to the influence of the busyness of leaders in the workplace and the impression that busyness left on others (Sherf et al., 2019). These viewpoints lay the foundation for the study to expand the research scenarios of busyness and deepen the understanding of the effects of busyness. The research expands and furthers the understanding of busyness and its effects on leaders and subordinates in both organizations.

Next, this study also contributes to perspective-taking literature and emphasizes the influence of perspective taking on the interaction between perceived leader busyness and interaction avoidance of followers. It highlights the negative influence that perspective taking may exert on individuals (Ku et al., 2015; Jing and Baiyin, 2016; Zhang and Tang, 2017). The research stresses that when followers have a high level of perspective taking, they are more likely to interpret information from the perspective of others and take actions beneficial to others. However, the study found that too much consideration of the leader may lead to reduced interaction with them, which can eventually lead to a negative employee evaluation; this would indicate the negative impact of perspective taking on the follower. As previous studies highlight, perspective taking often considers the situation of others from the perspective of individuals, but it can be misinterpreted and inaccurate. Such inaccurate interpretations of information may become a burden on followers (Campbell et al., 2014). Unlike previous studies that focused on the positive impact on dyadic relationships, we argue that perspective taking can bring negative results.

Finally, the research also contributes to the sensemaking theory and verifies the impact of information uncertainty on evaluations of individuals. In the workplace, not only will followers make sense of the behavior of leaders, but leaders will also make sense of the behavior of followers when making performance appraisals. Through empirical investigation, it is found that under the ambiguity and uncertainty of information, individuals are more inclined to make conservative, low-risk, and evasive decisions (Maes et al., 2018; Mai et al., 2018). This point also confirms that implicit behaviors in interpersonal interactions may also serve as clues for others to interpret information, which may impose negative effects.

### Practical Implications

This study has the following inspirations for practice: from the perspective of leaders, a high frequency of expressing a busy status to subordinates may create a sense of distance between followers, and followers may reduce their interactions with leaders. Followers with a high level of altruism may be more likely to consider the needs of their leaders because they assume that reducing communication with leaders will be helpful. Consequently, leaders need to express their busyness and maintain favorable communication with followers. Next, holistic data collection by leaders is vital to evaluate followers and to completely comprehend their personality, characteristics, and qualities. Doing so avoids subjective and one-sided judgments based on incomplete information. In addition, during the process of performance evaluation of followers, leaders are responsible for analyzing behavioral motivations or making judgments of employees based on performance.

Although perspective taking can promote mutual understanding most of the time, too much consideration of situations of others may cause certain pressure on followers and generate negative effects. Therefore, instead of over-accommodating situations of others, followers can adopt the method of direct communication to more accurately assess others’ needs.

### Limitations and Future Research Directions

In this study, individuals with high perspective taking responses to the busyness of their leaders were investigated. A series of discussions were conducted, mainly from the perspective of altruism. In the future, exploring different responsive behaviors, such as helping behaviors, could better develop the study of leader busyness. Future studies can also analyze the occasions for followers to interact with the leader. For example, when a follower is considerate and sensitive to other mood changes or has a tacit understanding with the leader (Zheng X. et al., 2019), s/he can make appropriate behavioral shifts. Therefore, it is essential to explore the different responses of employees based on the busyness of leaders. Moreover, it is possible that different personal characteristics or situational factors may work as contextual factors to influence the above relationships.

Next, this study focuses on the overall busy image of the leader. Although this concept has theoretical and practical significance, further research is necessary. “Real busyness” vs. “fake busyness” of leaders is mentioned in the article; this distinction is worth investigating. Related to this is the measurement of the busyness of leaders. Subjective evaluations of followers were adopted in this study, but busyness could be objectively measured by observing the schedule of leaders. In addition, the relationship between busyness and other leadership styles needs to be explored. For example, it would be useful to understand if busy leaders tend to engage in abusive behavior because of resource depletion or if they can still be benevolent. Conversely, we can also explore whether leadership style affects the judgment of followers of the busyness of leaders. For example, the exhibition of benevolent behavior may lead to a lowered perception of leader busyness, and the demonstration of abusive behaviors may result in an increase in the perception of leader busyness on the part of followers. There is ample room for further investigation.

Ultimately, although this study arrived at the conclusion that decreased interaction by followers would cause the leader to reduce a positive evaluation; this relationship is affected by a variety of factors. For example, followers with excellent working competence or outstanding performance would not be given a negative evaluation from leaders merely because of the reduced interactions. Therefore, the relationship between the interactive avoidance behavior of followers and leaders’ evaluations of followers tend to be affected by various elements. From this point of view, there is still room to explore leader busyness and the interactions between leaders and subordinates.

## Data Availability Statement

The raw data supporting the conclusions of this article will be made available by the authors, without undue reservation.

## Ethics Statement

The protocol for the current study was approved by the Institutional Review Board of Department of Administration Management at Huaqiao University. Written informed consent was obtained from all participants.

## Author Contributions

QH contributed to writing and editing the manuscript and approved the final manuscript as submitted. KZ generated the research idea with the theoretical development of the research, collected all the data, did the data analysis, edited the manuscript, and replied to the reviewer’s comments. Both authors contributed to the article and approved the submitted version.

## Conflict of Interest

The authors declare that the research was conducted in the absence of any commercial or financial relationships that could be construed as a potential conflict of interest.

## Publisher’s Note

All claims expressed in this article are solely those of the authors and do not necessarily represent those of their affiliated organizations, or those of the publisher, the editors and the reviewers. Any product that may be evaluated in this article, or claim that may be made by its manufacturer, is not guaranteed or endorsed by the publisher.

## Footnotes

1 Values above 0.90 for CFI indicate acceptable fit (Bentler, 1990), and values of 0.08 or less for RMSEA and SRMR are considered acceptable (Hu and Bentler, 1999).

2 Although perceived leader busyness and interaction avoidance were collected at the same time, we did not propose their direct relationships, but focused on the interactive effect. As noted in prior literature, when independent and outcome variables are collected at the same time, it is difficult to find an interactive effect (Podsakoff et al., 2012). Our study showed a stronger interactive effect, which may indicate the reliability of our results.
